# Lifespan-extending caloric restriction or mTOR inhibition impair adaptive immunity of old mice by distinct mechanisms

**DOI:** 10.1111/acel.12280

**Published:** 2014-11-26

**Authors:** Emily L Goldberg, Melissa J Romero-Aleshire, Kristin R Renkema, Melissa S Ventevogel, Wade M Chew, Jennifer L Uhrlaub, Megan J Smithey, Kirsten H Limesand, Gregory D Sempowski, Heddwen L Brooks, Janko Nikolich-Žugich

**Affiliations:** 1 Departments of Immunobiology and the Arizona Center on Aging, University of Arizona College of MedicineTucson, AZ, USA; 2 Department of Nutritional Sciences, College of Agriculture and Life Sciences, University of ArizonaTucson, AZ, USA; 3 Department of Physiology, University of Arizona College of MedicineTucson, AZ, USA; 4 Human Vaccine Institute, Duke UniversityDurham, NC, USA; 5 Arizona Cancer Center, University of Arizona College of MedicineTucson, AZ, USA

**Keywords:** anti-aging, cellular immunology, caloric restriction, longevity regulation, mouse models, T cell

## Abstract

Aging of the world population and a concomitant increase in age-related diseases and disabilities mandates the search for strategies to increase healthspan, the length of time an individual lives healthy and productively. Due to the age-related decline of the immune system, infectious diseases remain among the top 5–10 causes of mortality and morbidity in the elderly, and improving immune function during aging remains an important aspect of healthspan extension. Calorie restriction (CR) and more recently rapamycin (rapa) feeding have both been used to extend lifespan in mice. Preciously few studies have actually investigated the impact of each of these interventions upon *in vivo* immune defense against relevant microbial challenge in old organisms. We tested how rapa and CR each impacted the immune system in adult and old mice. We report that each intervention differentially altered T-cell development in the thymus, peripheral T-cell maintenance, T-cell function and host survival after West Nile virus infection, inducing distinct but deleterious consequences to the aging immune system. We conclude that neither rapa feeding nor CR, in the current form/administration regimen, may be optimal strategies for extending healthy immune function and, with it, lifespan.

## Introduction

As the world's population grows older, it is critical to understand comorbidities of aging and identify potential points of intervention to improve the quality of life in older adults. Despite vast improvements in health care, the aged population remains disproportionally vulnerable to infectious diseases and exhibits reduced vaccine efficacy (Gardner & Pabbatireddy, 2004). Age-related decline of immune function underlies this critical vulnerability.

Aging is associated with several changes in the immune system, collectively referred to as immune senescence [rev. in (Nikolich-Žugich, 2014)]. Thymic involution is the earliest sign of immune aging, starting in early adolescence (Yang *et al*., 2009; Chinn *et al*., 2012). This leads to decreased export of naïve T cells to the periphery, putting a stress upon the peripheral maintenance of the T-cell pools [rev. in (Sprent & Surh, 2011)]. Maintaining a balanced T-cell pool with adequate naïve and memory cell reserves is critical for protection against new and previously encountered pathogens, respectively [rev. in Nikolich-Žugich (2014)]. Yet, pathogen exposure throughout the lifetime converts many naïve T cells into memory cells, while the naïve T cells are themselves not being properly renewed. This leads to a declining naïve: memory T-cell ratio during aging. In addition, both CD4 and CD8 T cells have been shown to exhibit cell-intrinsic age-related defects in mice and humans (Nikolich-Žugich, 2014) some of which were associated with mortality from acute infection with Listeria monocytogenes (Lm) (Smithey *et al*., 2011), West Nile virus (WNV) (Brien *et al*., 2009) and Streptococcus pneumoniae (Shivshankar *et al*., 2011). This has been attributed (in part) to decreased proliferation of Ag-specific T cells (Po *et al*., 2002; Smithey *et al*., 2011), which compounds their defective ability to produce effector cytokines upon infection *in vivo* (Brien *et al*., 2009).

Calorie restriction (CR) is the only natural intervention known to extend lifespan (Masoro, 2009). CR is typically defined as a 20–40% reduction in caloric intake compared to *ad libitum* feeding, providing a limitation in calories in the absence of malnutrition (essential micronutrients are provided at 100% level). Broad benefits of CR in aging rodents include improved cardiovascular, neurological, muscular, and skeletal health [rev in (Fontana *et al*., 2010)]. CR was shown to impact immune cell function [rev. in Nikolich-Žugich & Messaoudi (2005)] by preserving thymic cellularity during aging (Yang *et al*., 2009) and perhaps consequently contributing to an increased proportion of naïve CD8 T cells later in life (Yang *et al*., 2009). Whether CR improves immune function against live microbial challenge is incompletely understood. The few studies using pathogen challenge *in vivo* showed that CR may increase susceptibility to infectious diseases (Sun *et al*., 2001; Gardner, 2005; Kristan, 2007), despite its ‘beneficial’ impact upon steady-state immunity and *in vitro* immune response. Notably, the infection models used in these studies resulted in death of CR mice early postinfection, reflecting defects in the innate immune system. Also, the primary infection model used in CR mice was influenza A virus (IAV) (Effros *et al*., 1991; Gardner, 2005), which in mice induces wasting and significant weight loss, potentially confounding the survival in mice that have no spare weight to lose. Overall, it remains unclear whether CR alters the ability of the adaptive immune system to protect mice from infection *in vivo*.

Rapa was recently used to extend lifespan in genetically heterogeneous mice, even when initiated late in life (Harrison *et al*., 2009; Zhang *et al*., 2013). This immune suppressant specifically inhibits the mTORC1 signaling complex, which has long been considered a regulator of lifespan (Johnson *et al*., 2013). This landmark discovery highlighted the possibility of using pharmaceutical interventions to extend lifespan in mammals. To date, only a single study has investigated the effect of rapa feeding on immune protection in old mice (Hinojosa *et al*., 2012) and demonstrated that rapa-treated old mice exhibited improved survival following pneumococcal infection. Mice die from pneumococcal pneumonia infection within 4 days of infection, before the development of measurable Ag-specific responses, so these authors (Hinojosa *et al*., 2012) could not evaluate Ag-specific immunity, leaving the question of how adaptive immunity in old mice is altered by lifespan-extending rapa treatment unresolved.

This highlights a critical question: Do lifespan-extending interventions only extend lifespan or do they also extend ‘healthspan’ (the length of healthy, high quality lifespan)? Here, we approached this question by interrogating the impact of CR and rapa feeding on the aging immune system under the conditions of lethal infectious challenge. We report that rapa and CR treatments have opposite effects on thymic cellularity, peripheral homeostasis, and subset balance of naïve and memory T-cell subsets. Perhaps most importantly, CR mice exhibited significantly increased mortality from WNV infection and poor Ag-specific T-cell function, with the mortality of rapa-treated group trending lower, but not reaching statistical significance. We conclude that thorough examination of immune function is essential to evaluate whether lifespan extension interventions can improve healthspan and that both rapa and CR, during prolonged administration, impose additional immunological defects during aging.

## Results

To determine how lifespan-extending interventions impact the aging immune system, we compared the effects of lifelong CR or 60-day rapa feeding to age-matched control male C57BL/6 mice at least 18 months old at the time of analysis. These treatment regimens were chosen based on their ability to increase median and maximal lifespan in mice (Harrison *et al*., 2009; Masoro, 2009). We are aware that the differences in length of treatment and the age at which treatments were initiated may have influenced our observations and have tried to highlight those considerations accordingly. Initial gross observations revealed no obvious physical detriments with either treatment compared to controls. Mice on rapa exhibited slightly reduced body weight compared to age-matched controls (Fig. S1A), probably due to potent lipolytic effect of rapa (Harrison *et al*., 2009), although this effect is known to depend on the duration of treatment (Fang *et al*., 2013). CR mice exhibited the expected decline in body weight and food intake compared to both control and rapa-treated old mice (Fig. S1A, B).

**Figure 1:**
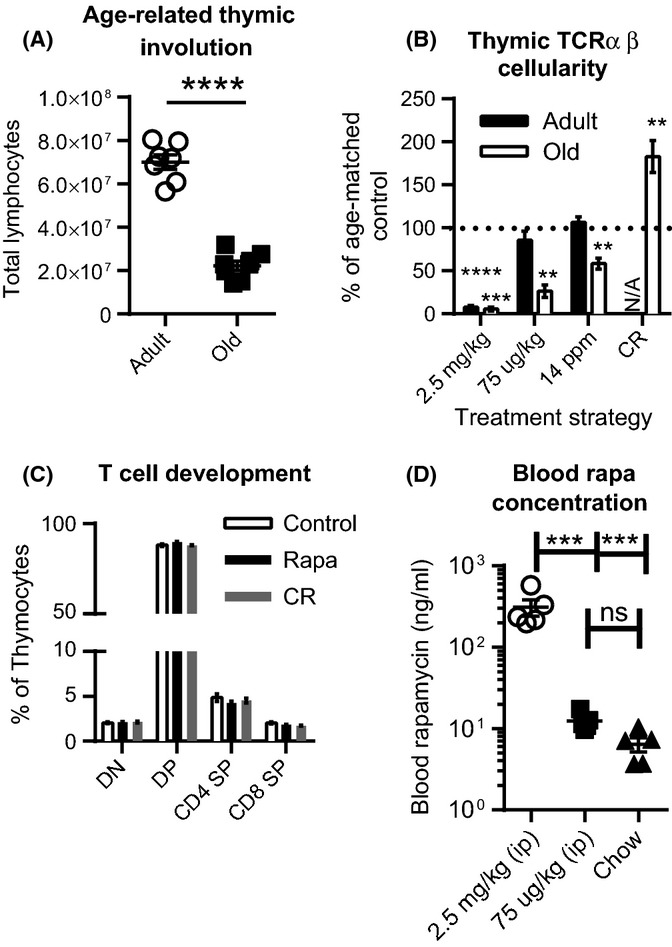
Rapamycin and calorie restriction have different effects on thymic cellularity. Mice were placed on rapa for 2 months or on CR for life. (A) Untreated A and O mouse thymus cellularity. (B) Thymi were harvested from A and O mice from the indicated treatment groups and total thymocytes enumerated. (C) Thymus single-cell suspensions were analyzed for thymocyte development based on CD4 and CD8 expression. (D) Whole blood rapa concentration was measured in O mice being treated with the indicated doses and routes. Data represent the combination of 2 independent experiments, with *n* = 2–5 mice per replicate. (B) Thymic cellularity is represented as % of age-matched controls that were harvested at the same time, to control for different dosing routes. 2.5 mg kg^−1^ and 75 μg kg^−1^ were given as daily i.p. injections. 14 ppm was the concentration of rapa in rodent chow, as described in the experimental procedures section. Statistical differences were calculated by Student's *t*-tests compared to the cellularity of age-matched, control-treated mice. (C-D) Statistical differences were calculated by 1-way ANOVA with Bonferroni's post-tests. *P<0.05; **P<0.01; ***P<0.001.

We next determined the effects of each treatment on T-cell development in the thymus. A well-documented thymic involution with aging was found (Fig.1A), and in agreement with previous reports (Chen *et al*., 1998), CR was able to partially mitigate this loss of thymic cellularity with age (Fig.1B, shown as the % of cellularity from age-matched control mice, harvested at the same time; absolute counts provided in Table S1). However, both adult (A) and old (O) mice treated with a high dose of rapa (2.5 mg kg^−1^ day^−1^ i.p.) exhibited severe loss of thymic cellularity. In mice fed rapa-containing chow, drug blood levels were substantially lower compared to high dose (Fig.1D), and only the O mice showed significant reduction of thymic cellularity compared to controls (Fig.1B). Thymocyte subset distribution did not appear altered by rapa at any dose, based on the expected and equal distribution of CD8-4- double-negative (DN), CD8 + 4 + double-positive (DP), and the CD4 and CD8 single-positive subsets between groups (Fig.1C), each of which marks a discrete stage of thymocyte maturation. This suggested that the individual treatments did not induce specific developmental blocks, impacting all subsets symmetrically, or perhaps impacting only some of the earliest precursors (e.g., HSC or early T-cell precursors). We did not further investigate high-dose rapa treatment, because it is not used to extend longevity and due to its highly deleterious effects on the thymus. We focused upon the rapa chow, which in our hands produced rapa blood levels similar to rapa levels in transplant patients taking sirolimus (Mahe *et al*., 2005), although in genetically heterogeneous mice, rapa feeding results in blood levels 3× than that of patients (Harrison *et al*., 2009).

At this point, we considered the possibility that CR and rapa treatments may alter hormones known to impact the thymus. Rapa feeding has been reported to accelerate testicular degeneration in O male mice (Wilkinson *et al*., 2012), and androgens are known to significantly impact thymocyte survival (Min *et al*., 2006; Radojevic *et al*., 2007). Therefore, we measured serum testosterone levels in control and rapa age-matched O mice. Mice treated with rapa exhibited significantly increased serum testosterone levels (Fig. S2). This change could modulate and pronounce the decreased thymic cellularity, as increasing testosterone levels during puberty are associated with thymus involution (Chinn *et al*., 2012), and castration early in life preserves thymic cellularity (Radojevic *et al*., 2007), although it is also clear that sex steroids alone do not causally and singlehandedly cause thymic involution (Chinn *et al*., 2012; Griffith *et al*., 2012). We conclude that rapa and CR have significant and opposite effects on the thymus and T-cell development.

**Figure 2:**
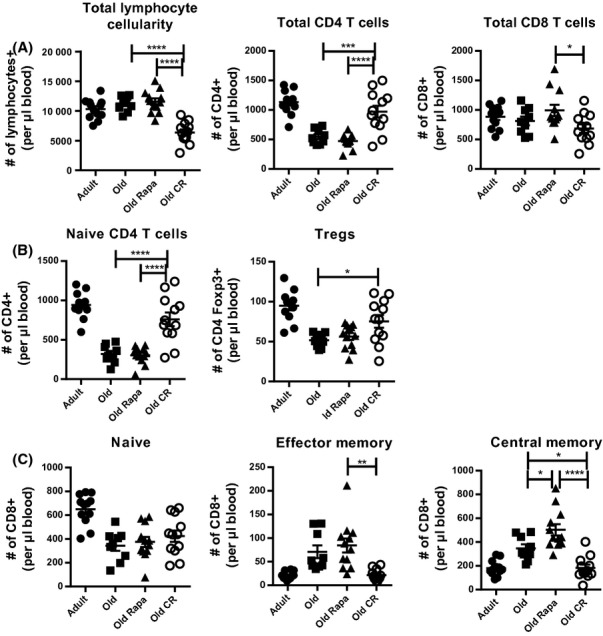
Rapamycin and calorie restriction differently alter peripheral T-cell subset absolute counts. Mice were bled after 2 months of rapa feeding when O mice were 18 months old. (A) Total lymphocyte, total CD4, and total CD8 T-cell absolute counts in the blood. (B) Absolute counts of N (CD44^low^CD62L^hi^) and regulatory (Foxp3^+^) CD4 T cells in the blood. (C) N, CM, and EM cells were identified enumerated based on CD44 and CD62L expression. Data are shown as mean ± SEM and are representative of 3 independent cohorts. Statistical differences between O groups were calculated by 1-way ANOVA with Bonferroni post-tests. Adults are shown to provide a reference point and were not included in statistical analyses. *P<0.05; **P<0.01; ***P<0.001.

We next examined whether the above alterations in thymic cellularity affected peripheral T-cell pools. We first enumerated blood T-cell subsets, finding the expected general lymphopenia in O mice on CR, which was not caused by T-cell (total CD4 or CD8) depletion (Fig.2A). Within the CD4 T-cell compartment, O CR mice had significantly more naïve (N; CD44^low^CD62L^hi^) CD4 T cells and regulatory T cells (Tregs, FoxP3+) compared to controls (Fig.2B). Despite an increased relative frequency of N CD8 T cells in CR mice compared to controls (Fig. S3) absolute numbers of N CD8 T cells in the blood were similar in all three groups of O mice (control, rapa, CR, Fig.2C). CR mice exhibited lower cell counts of CD8 central memory (CM, CD44^hi^CD62L^hi^) and effector memory (EM, CD44^hi^CD62L^low^) cells, whereas rapa had no effect upon these subsets. Overall, we conclude that in O mice CR significantly alters blood T-lymphocyte population abundance during aging.

**Figure 3:**
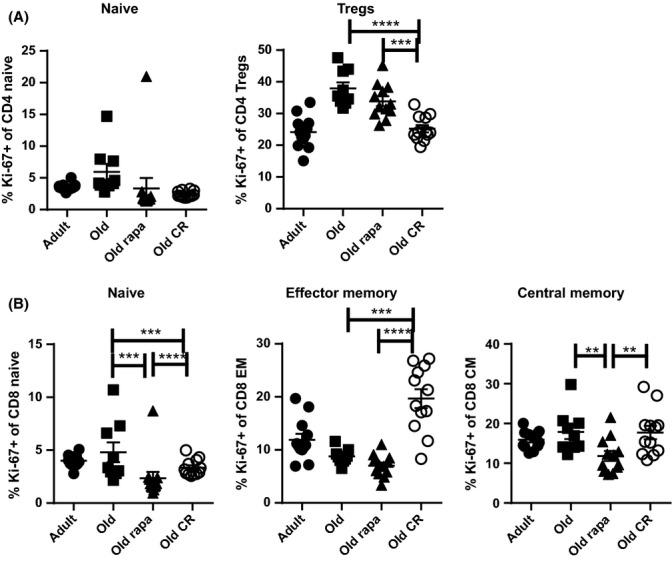
Rapamycin and calorie restriction differently alter T-cell subset steady-state proliferation. Steady-state proliferation was evaluated by Ki-67 staining within naïve, effector memory, and central memory CD8 T-cell subsets. (A) The frequency of cells within the naïve CD4 T-cell subset or within regulatory Tregs that are Ki-67^+^. (B) The frequency of cells within the indicated CD8 T-cell subsets that were Ki-67^+^. Data are shown as mean ± SEM and are representative of 3 independent cohorts. Statistical differences between O groups were calculated by 1-way ANOVA with Bonferroni post-tests. Adults are shown only to provide a young reference point and were not included in statistical analyses. *P<0.05; **P<0.01; ***P<0.001.

We also examined whether the above treatments impacted homeostatic behavior of peripheral T-cell subsets, by testing cycling rates of these cells by measuring Ki-67, a nuclear protein expressed by actively proliferating cells, within each subset. While N CD4 T-cell steady-state proliferation was not altered by rapa or CR, significantly fewer Tregs were in cycle in CR O mice (Fig.3A) compared to O controls. In all three groups, very few N CD8 T cells were Ki-67^+^ and this was further reduced by rapa (Fig.3B). Surprisingly, we observed a substantial increase in the abundance of Ki-67+ EM CD8 T cells in CR mice compared to both control and rapa groups (Fig.3B). Therefore, the few EM cells (Fig2C) in CR-treated mice were highly proliferative, suggesting that these cells in CR mice turn over vigorously. It remains to be seen whether this turnover is stimulated by homeostatic (e.g., relative IL-15 abundance) or other, perhaps antigen-driven, mechanisms. Rapa treatment decreased the abundance of the Ki-67+ CM subset compared to both control and CR, suggesting that this subset could be pushed to quiescence by mTOR inhibition. Although we had not intended to directly compare metabolic signaling/requirements in T-cell subsets, the different effects of each treatment on different subsets supports the hypothesis that distinct T-cell populations have different metabolism and will thus respond differently to treatment. Overall, the impact on the fraction of proliferating cells at steady state was directly opposite to the abundance of the given subset; in other words, abundant subsets tended to turn over less vigorously in O treated mice, and these values were similar to untreated A mice. It will be important to examine the impact of rapa and CR upon cell survival and/or trafficking. Overall, our data indicate that rapa and CR induce distinct differences in peripheral CD8 T-cell subset maintenance.

Because rapa feeding and CR exerted different effects on absolute counts of T-cell subsets in the periphery, we asked whether the changes in representation of cells undergoing steady-state proliferation were caused by changes in absolute numbers of cycling cells in each subset. Old N CD4 and CD8 T cells were sensitive to rapa's antiproliferative effect, as judged by a decreased number of such cells expressing Ki-67 (Fig. S4A, B) compared to O control cells. However, numbers of Ki-67+ CD4 Treg cells and CD8 effector memory cells did not change in O mice regardless of rapa treatment (Fig. S4A, B). This is in contrast to significant changes in the frequencies of Ki-67+ cells within these subsets (Fig.3A, B), suggesting that rapa treatment decreases the nonproliferating fraction of these of Tregs and CD8 EM cells, by redistribution to other organs or by true depletion. Conversely, although the frequency of CD8 CM Ki-67+ cells was decreased by rapa feeding, this was not due to a decline in the number of cells within this subset (Fig. S4B). We conclude that naïve T cells appear particularly susceptible to the rapa-mediated cell cycle inhibition; the significant increase in the fraction of proliferating Treg and CD8 effector memory in O CR mice is likely due to the relative overrepresentation of these cells (Fig. S4B, C, respectively).

**Figure 4:**
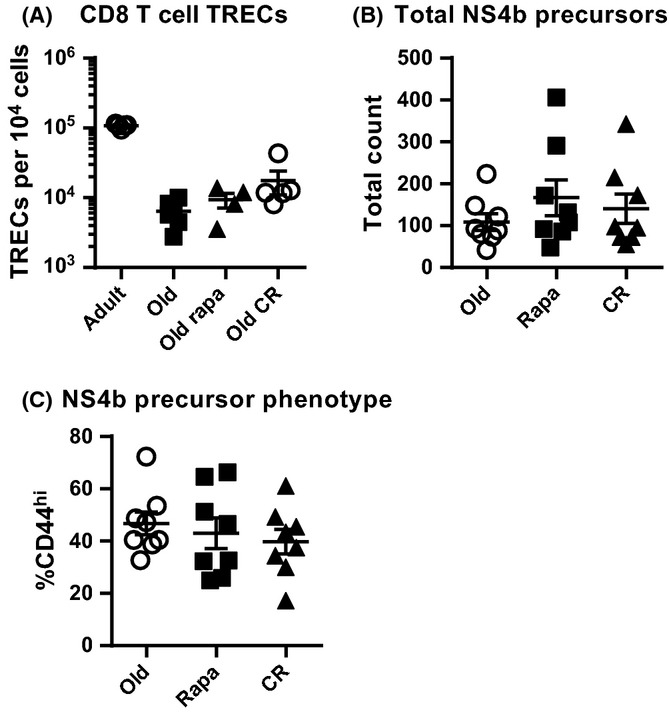
Rapamycin and calorie restriction do not alter thymic output. Spleen and superficial lymph nodes were harvested and analyzed for TRECs and Ag-specific CD8 T-cell precursors. (A) CD8 T-cell TRECs were quantified. Data shown are pooled from 2 independent harvests, each with *n* = 2–3 mice per group in each replicate. (B) CD8 T-cell precursors specific for the immunodominant WVN epitope NS4b were quantified. (C) Memory phenotype within the NS4b-specific CD8 T-cell precursors was measured. (B-C) Data are shown as mean ± SEM and are combined from 3 independent harvests, each with *n* = 2–3 mice/group at each harvest. (A) Statistical differences between O groups were calculated by nonparametric Kruskal–Wallis test with Dunn's multiple comparison test. Adults are shown only as a reference point and were not included in statistical analyses. (B, C) Statistical differences were calculated by 1-way ANOVA with Bonferroni's post-test

It was surprising that higher thymocyte counts found in O CR mice failed to translate into increased numbers of naïve CD8 T cells in the periphery. To determine whether the altered thymic cellularity in each treatment group also changed thymic output of CD8 T cells, we quantified T-cell receptor excision circles (TRECs; a by-product of TCR gene rearrangement that remain in the cell episomally, used to estimate thymic output of cells (Sempowski *et al*., 2002) from enriched CD8 T cells in the spleen and superficial lymph nodes. In contrast to previous reports on CD4 T cells in CR (Yang *et al*., 2009), we found no difference in CD8 T-cell TRECs in rapa or CR O mice compared to age-matched controls (Fig.4A). Therefore, the CR-mediated improvement in thymic cellularity did not result in increased CD8 T-cell export to the periphery.

To confirm and extend these observations, we directly quantified antigen-specific CD8 T-cell precursors from the spleen and superficial lymph nodes of unimmunized mice (Moon *et al*., 2007). Again, CR and rapa mice had the same number of CD8 cells specific for the West Nile virus (WNV) class I immunodominant epitope, NS4b (Brien *et al*., 2009) as did the control O mice (Fig.4B), despite dramatic differences in thymic cellularity (Fig.1B). In aging, many naïve precursors become CD44^hi^ virtual memory cells despite never encountering antigen, becoming functionally inferior to truly naïve CD44^low^ precursors [rev. in Nikolich-Žugich (2014)]. NS4b virtual memory precursors in CR and rapa mice were numerically comparable to untreated O controls (Fig.4C). Thus, the equal numbers and the equal content of memory-phenotype precursors in all groups were consistent with the equal TRECs content.

**Figure 5:**
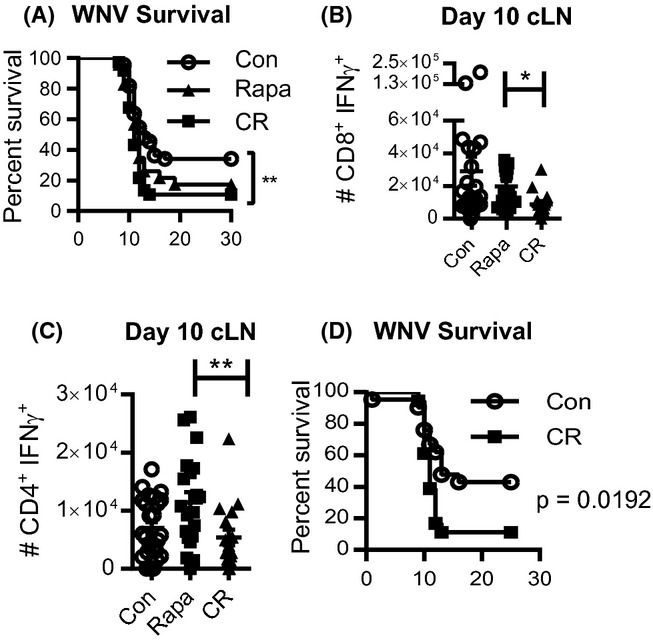
Calorie restriction, but not rapamycin, increases WNV mortality in old mice. Mice were infected with 10^3^ pfu WNV s.c. and tracked 30 days after infection or sacrificed on day 10 postinfection for Ag-specific T-cell analysis. (A) Survival after WNV infection in O, O rapa, and O CR mice. (B) Total number of NS4b-specific CD8 T cells that produce IFNγ upon *ex vivo* peptide stimulation. (C) Total number of CD4 T cells that produce IFNγ after *ex vivo* peptide stimulation with a pool of CD4 epitopes as indicated in the materials and methods. (D) Adult control and CR mice were infected with 10^3^ pfu WNV and tracked for survival. (A) Data shown are the combination of 3 independent experiments (*n* = 9–12 mice/group in each experiment). Statistical differences were calculated by log-rank test. (B, C) Data are shown as mean ± SEM and are representative of 2 independent experiments each containing *n* = 12 mice/group. Statistical differences were calculated by nonparametric Kruskal–Wallis test with Dunn's multiple comparison test. (D) Data shown are the combination of 2 independent experiments with *n* = 19 mice/group total. Statistical differences were calculated by log-rank test. *P<0.05; **P<0.01; ***P<0.001.

Finally, we asked whether the treatment by rapa or CR alters immune protection against infection, by infecting O control, O rapa, and O CR mice with 1000 pfu WNV s.c., a dose that killed >60% of O control mice. CR mice were significantly more susceptible to lethal WNV meningoencephalitis compared to O controls (*P* = 0.0029) (Fig.5A). Rapa mice exhibited lower, but not statistically significant, survival compared to controls (*P* = 0.1015), a point discussed below. Because both CD4 and CD8 T cells are critically important for protection against WNV (Sitati & Diamond, 2006; Brien *et al*., 2009), we also evaluated WNV-specific T-cell responses. In the brain's draining lymph nodes (cervical lymph nodes, cLN), CR mice contained significantly fewer CD8^+^ and CD4^+^ T cells able to produce IFNγ, a critically important antiviral mediator, upon *ex vivo* peptide stimulation (Fig.5B, C). Importantly, we also tested whether the increased WNV mortality with CR was age-specific. We found that A mice on CR diet for 2 months also exhibited significantly increased mortality after WNV infection compared to age-matched controls (Fig.5D). Overall, CR mice exhibited significantly impaired T-cell function, consistent with the increased mortality in CR mice. This is consistent with recent findings that the enormous metabolic expenditure necessary to fuel strong primary effector T-cell responses cannot tolerate dampening in either total calories (this paper) or the mTOR axis activity (Goldberg *et al*., 2014).

## Discussion

We have previously shown that age-related defects in CD8 and CD4 T cells are associated with increased WNV-mediated mortality in mice (Brien *et al*., 2009). Therefore, when an immunosuppressive drug was used to extend lifespan in mice (Harrison *et al*., 2009), we wondered whether this could have deleterious consequences to the already dysfunctional old T cells, particularly because we found that low-dose rapa treatment results in increased mortality in A mice infected with WNV (Goldberg *et al*., 2014). How rapa treatment would compare to the long-term lifespan-extending intervention, CR, was not clear. CR has been hypothesized to work, in part, by decreasing mTOR signaling (Blagosklonny, 2010). However, increasing evidence suggests a more complex interaction, whereby multiple pathways are likely altered by CR to increase longevity.

We found a surprising and contrasting effect of CR and rapa on thymic cellularity. The sensitivity of the old thymus to very low doses of rapa was striking. One could speculate that this could be mediated by hormonal changes induced by rapa, as we found increased serum testosterone levels in the O rapa group, consistent with findings that castration early in life preserves thymic cellularity (Radojevic *et al*., 2007). However, increased testosterone was somewhat unexpected given a previous report that rapa increases testicular degeneration in O mice (Wilkinson *et al*., 2012), although in that study, testicular degeneration was defined based on the effects on spermatogenesis, which is independent of testosterone production (Cheng & Mruk, 2010); it cannot be excluded that elevated testosterone could be leading to testicular atrophy as well. Alternativelyn, Notch-induced activation of the PI3K/AKT/mTOR signaling axis is indispensable for pre-T-cell glycolytic metabolism and viability (Ciofani & Zuniga-Pflucker, 2005), so rapa could drastically perturb the actively dividing T-cell precursors in an old thymus. Furthermore, rapa treatment was shown to dysregulate thymocyte development *in vitro* by decreasing proliferation and the transition from the CD8-CD4- to the CD8 + CD4 + stage (Kelly *et al*., 2007). We therefore speculate the deleterious effects of rapa on the aging thymus are likely due to metabolic and cell survival defects, an issue that will require additional research.

Beyond numerical reduction, there were no obvious specific blocks in T-cell development among the four major thymocyte subsets. One caveat to this interpretation is that we harvested tissue in the steady state, after a 60-day treatment. Early effects of treatment may be quite different (Fang *et al*., 2013). Indeed, at the onset of CR there is rapid thymic cellularity loss, with subsequent restoration and ultimately sustained cellularity increase during aging (Chen *et al*., 1998). Analogous studies using rapa have not been performed and would be highly informative. The decreased thymic cellularity in rapa mice could also be due to impaired emigration from the bone marrow, or decreased thymic entry of thymocyte progenitors. Hematopoietic stem cells (HSCs) exhibit depressed mTOR signaling to maintain quiescence. They also lose the propensity to develop into lymphoid lineage, so additional mTOR inhibition could further impair early T-cell precursor generation from HSC, contributing to thymic defects. Additional experimentation is needed to address either of these possibilities.

CR has been known to preserve a ‘young’ or naïve phenotype in the aging immune system (Weindruch *et al*., 1982; Effros *et al*., 1991; Messaoudi *et al*., 2006). Among our most surprising findings was that the ‘younger’ phenotype of the thymus or the peripheral T cells of CR mice did not translate to increased immune responses or better protection during infection. While the peripheral CD8 T-cell pool had an increased proportion of naïve cells (similar to our prior studies in monkeys (Messaoudi *et al*., 2006) the absolute number of naïve CD8 T cells was not increased in O CR mice compared to controls and that was consistent with an unaltered spleen and lymph node TRECs content. We would predict that rapa's deleterious effect on the thymus would eventually impose significant strain on the peripheral CD8 T-cell repertoire in the course of long-term lifespan extension treatment.

Previous studies have shown that CR T cells are more responsive to mitogen stimulation *in vitro* (Weindruch *et al*., 1982; Effros *et al*., 1991; Messaoudi *et al*., 2006). Given that the percentage of naïve T cells, which are the best responders to mitogenic stimulation, is significantly higher in spleens of CR mice compared to *ad libitum* fed controls, these results, which did not correct for such differences, can easily be explained by unequal composition of the responding cells. It was not known whether the above ‘improvement’ would translate to (i) better T-cell responses during infection and (ii) improved immune protection *in vivo*. We found the opposite to be the case during WNV infection: CR mice contained significantly fewer CD8 and CD4 T cells able to produce IFNγ in response to the virus, and this was associated with increased susceptibility to disease. This was similar to previous reports on CR mice infected with influenza virus (Gardner, 2005). However, unlike flu, mortality from WNV infection is observed on days 9–15 postinfection, and it is the failures in adaptive immunity that ultimately cause increased susceptibility to WNV in O mice (Brien *et al*., 2009). Additionally, the increased number of Tregs in O CR mice (Fig.2B) could also limit the expansion of responding CD4 and CD8 T cells during WNV infection (Lanteri *et al*., 2009). Therefore, our data demonstrate that in addition to innate defects (Ritz *et al*., 2008), O CR mice also exhibit adaptive immune system defects, with a potential to lead to increased mortality after acute infection. It would be of interest to know whether the immune defects in O CR mice are permanently programed, or whether they can be reversed by refeeding prior to infection (Clinthorne *et al*., 2010). Additional studies are required to test whether refeeding or alternate-day fasting can improve survival after infection, while retaining the beneficial effects of CR. Furthermore, how CR or rapa alters T-cell memory and vaccine efficacy have not been studied in aging, particularly if initiated after a substantial fraction of immune memory has been formed. As memory responses are a critical aspect of the adaptive immune system, and rapa has been shown to enhance CD8 T-cell memory, it will be of interest to investigate how these interventions impact T-cell memory.

CR is thought to operate, at least in part, by decreasing mTORC1 signaling. However, it is increasingly clear that other pathways contribute to the overall health benefits observed in CR animals, likely including insulin/insulin-like growth factor (IGF) signaling and AMP-activated protein kinase (AMPK) activation, because O mice undergoing life extension treated with rapa do not replicate benefits previously reported for CR (Wilkinson *et al*., 2012; Neff *et al*., 2013). The likelihood that CR operates by many mechanisms in addition to decreased mTORC1 signaling is further supported by a recent publication showing that the two interventions have different metabolic outcomes in aged mice (Miller *et al*., 2014). In the immune system, our data suggest that CR may function via distinct, and perhaps entirely different, mechanism(s) from rapa, as CR and rapa exhibited contrasting effects in almost every analysis we performed. However, this issue requires additional research, including accounting for the critical caveat that CR was a lifelong intervention, whereas the rapa feeding began late in life.

While rapa and CR differentially affected the aging immune system, changes induced by these agents were not beneficial and they each had deleterious consequences to immunity. The negative effect of CR on WNV infection susceptibility was clear. Rapa-treated groups showed lower survival compared to controls, but the latter did not reach statistical significance. However, we would argue that our model was not optimally powered to statistically validate the subtler deleterious effects of rapa. This is because most of our untreated O mice did not survive WNV infection, providing us with a very narrow window within which to score increased susceptibility to WNV. In A mice rapa-induced T-cell immunity defects correlated with lethal infection outcome (Goldberg *et al*., 2014). On the basis of that and on the above results, we conclude that neither CR nor rapa, in the doses and administration regimens used in our study, may be ideal interventions for promoting healthspan-associated longevity, stressing the need for better and more successful rapalogues/substances manipulating the mTOR pathway.

## Experimental procedures

### Mice

Male A (16 weeks old) and O (16–18 months old) C57BL/6 mice were purchased from Jackson Laboratories (Bar Harbor, ME) and the National Institute on Aging (Charles River), respectively. Mice were housed in sterile caging under specific pathogen-free conditions. All experimental procedures were conducted with approval from the University of Arizona Institutional Animal Care and Use Committee.

### Calorie restriction

Old CR male mice were purchased from the NIA aged rodent colony. CR is initiated at 14 weeks old, restricting 10% of food intake compared to *ad libitum* controls, progressing to 25% at 15 weeks and 40% at 16 weeks, the level (3 g daily, NIH-31 fortified formula) maintained for life. CR mice were purchased at the oldest available age and then housed at the University of Arizona until 18 months old. For A CR studies, mice were infected at 25 weeks of age (fully weaned on to the CR diet 2 months prior to infection).

### Rapamycin treatment

Treatments with high-dose (LC Laboratories, Woburn, MA, USA; 2.5 mg kg^−1^; daily i.p. injection) and low-dose injections (LC Laboratories; 75 μg kg^−1^ daily) or low-dose chow (LC Laboratories, encapsulated in chow at 14 ppm by Southwest Research Institute, San Antonio, TX, USA, formulated as described previously [Harrison *et al*., 2009)] rapa were initiated 2 months prior to analysis or manipulation. All immunological analyses and manipulations were performed in mice at least 18 months old. Blood rapa concentrations were measured by HPLC–mass spectrometry exactly as described previously (Kusne *et al*., 2013). Rapa concentrations were verified in paired samples by Dr. Martin Javors (UT Health Science Center, San Antonio, TX, USA) by independent analysis (Harrison *et al*., 2009).

### Serum hormone measurements

Sera were collected from retro-orbital bleeds, following clotting on ice for 30 min and were stored at −80 °C until analysis, with no freeze/thaw cycles prior to analysis. Mouse testosterone levels were quantified by ELISA (Enzo Life Sciences, Farmingdale, NY, USA) according to the manufacturers’ instructions.

### Flow cytometry

Blood samples were collected by retro-orbital bleed. Tissues were harvested after isoflurane overdose followed by cardiac puncture. Tetramer and flow cytometry staining and *ex vivo* peptide stimulation for intracellular cytokine staining were performed as described (Smithey *et al*., 2011). For thymocyte gating, non-αβ T-cell precursors were excluded with a dump gate that included the following cell surface markers: γδ TCR, NK1.1, CD19, and B220. Samples were fixed and permeabilized using the Foxp3 buffer kit (eBioscience, San Diego, CA, USA) followed by intracellular staining. Samples were collected on a custom LSR Fortessa flow cytometer (BD Biosciences, San Jose, CA, USA) and analyzed using flowjo software (Tree Star, Ashland, OR, USA). All antibodies were purchased from eBioscience, BD, and Invitrogen. D^b^-NS4b tetramer was provided by the NIH tetramer core facility (Emory University, Atlanta, GA, USA).

### Quantitation of T-cell receptor excision circles and CD8 T-cell precursors

Total CD8 T cells were enriched from spleen and superficial lymph nodes using a negative selection kit (Miltenyi), normalized for cell counts, pelleted, snap frozen, and stored at −80 °C until analysis. T-cell receptor excision circle (TREC) quantitation was performed as described (Sempowski *et al*., 2002). CD8 T-cell precursors were enriched from spleen and superficial lymph nodes as described previously (Rudd *et al*., 2011).

### Infection and vaccination

Mice were infected s.c. with 1000 pfu WNV strain 385-99 generously provided by Dr. Robert Tesh (University of Texas Medical Branch, Galveston). CD8 T-cell Ag-specific responses studied were restricted to the CD8 immunodomonant epitope, NS4b; CD4 T-cell responses were measured as the pooled response to 4 CD4 immunodominant epitopes: E347, 2066, E641, and 1616, as in (Brien *et al*., 2009). *Ex vivo* stimulation was performed in the presence of Brefeldin A, for 5 h at 37 °C.

### Data analysis

Statistical analyses were performed using graphpad prism software (GraphPad, La Jolla, CA, USA), as indicated in the text and legends. Data were evaluated for normal distribution, and parametric or nonparametric tests were used, as appropriate. *P* < 0.05 was considered significant for all analyses.

## Abbreviations

A: adult mice, 4 months old
CM: central memory T cells, CD62L^hi^44^hi^
CR: calorie restriction
EM: effector/effector memory T cells, CD62L^lo^44^hi^
mTOR: mechanistic (mammalian) target of rapamycin
N: naïve T cells, CD62L^hi^44^lo^
O: old mice, >18 month old
Rapa: Rapamycin
Treg: regulatory T cells, FoxP3+

## Supporting Information

**Fig. S1** Rapamycin and calorie restriction alter mouse body weight and food consumption. **Fig. S2** Rapamycin increases serum testosterone in old mice. **Fig. S3** Calorie restriction alters peripheral CD8 T-cell subset frequencies in old mice. **Fig. S4** Naïve T cells exhibit unique sensitivity to inhibition of cell proliferation by rapa feeding.

Table S1 Absolute thymocyte counts.
