# Supplementary figures and images for: Lifespan-extending caloric restriction or mTOR inhibition impair adaptive immunity of old mice by distinct mechanisms
Source: Aging Cell. 2014 Nov 26;14(1):130–8. doi: 10.1111/acel.12280 (PMC4326902; doi:10.1111/acel.12280)

# Supplemental Figure 1

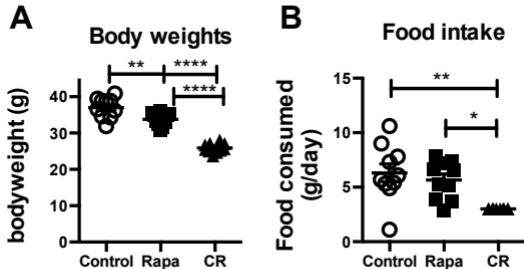

## Supplemental Figure 2

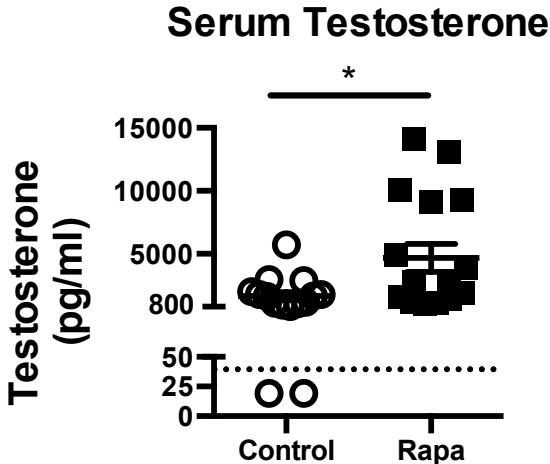

# Supplemental Figure 3

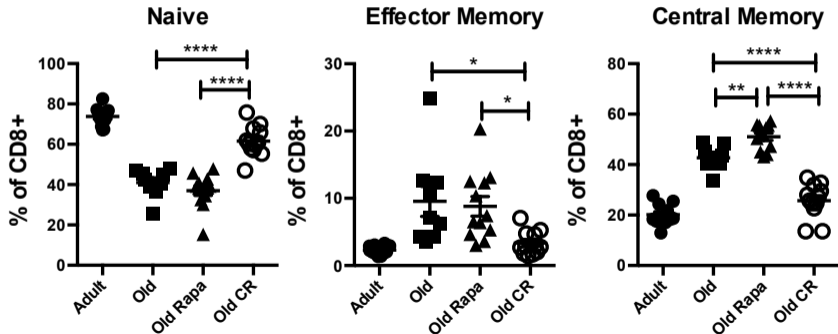

# Supplemental Figure 4

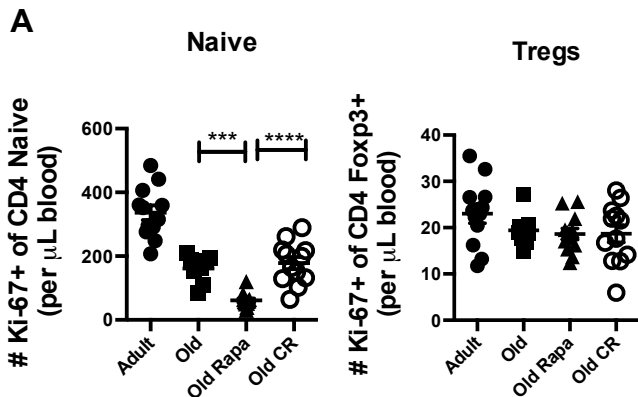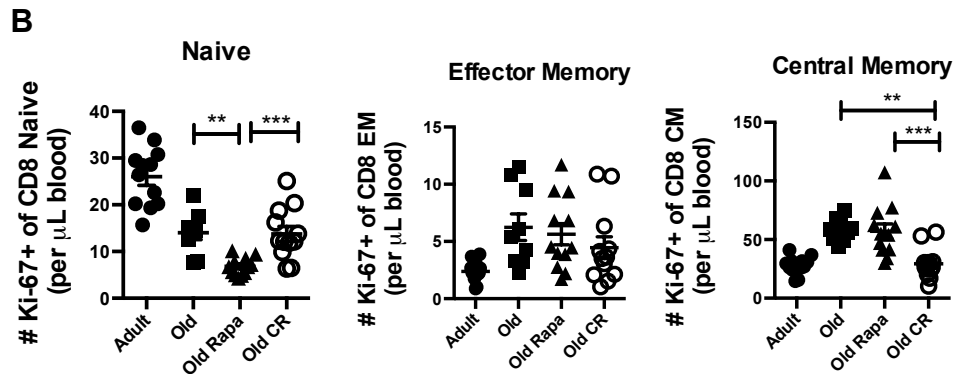

Supplement: Supplementary file 1 — Fig. S1 Rapamycin and calorie restriction alter mouse body weight and food consumption. Fig. S2 Rapamycin increases serum testosterone in old mice. Fig. S3 Calorie restriction alters peripheral CD8 T-cell subset frequencies in old mice. Fig. S4 Naïve T cells exhibit unique sensitivity to inhibition of cell proliferation by rapa feeding. [file acel0014-0130-sd1.pdf]
