# Supplementary material for: Lifespan-extending caloric restriction or mTOR inhibition impair adaptive immunity of old mice by distinct mechanisms
Source: Aging Cell. 2014 Nov 26;14(1):130–8. doi: 10.1111/acel.12280 (PMC4326902; doi:10.1111/acel.12280)
Supplement: Table S1 — Absolute thymocyte counts. [file acel0014-0130-sd2.docx]

|  | Control | Rapa chow | CR |
| --- | --- | --- | --- |
| Adult | 7.009x10^7^  ± 3.377x10^6^ | 7.326  ± 5.678 x10^6^ |  |
| Old | 1.295x10^7^  ± 2.180x10^6^ | 2.544x10^6^  ± 5.148x10^5^ | 1.896x10^7^  ± 1.935x10^6^ |

Supplemental Table 1. Absolute thymocyte counts.
